# Effects of a food-based intervention on markers of micronutrient status among Indian women of low socio-economic status

**DOI:** 10.1017/S000711451400419X

**Published:** 2015-02-13

**Authors:** Sarah H. Kehoe, Harsha Chopra, Sirazul A. Sahariah, Dattatray Bhat, Renuka P. Munshi, Falguni Panchal, Stephen Young, Nick Brown, Dnyaneshwar Tarwande, Meera Gandhi, Barrie M. Margetts, Ramesh D. Potdar, Caroline H. D. Fall

**Affiliations:** 1 Medical Research Council Lifecourse Epidemiology Unit, University of Southampton, Southampton, UK; 2 Centre for Study of Social Change, Mumbai, India; 3 Diabetes Unit, King Edward Memorial Hospital Research Centre, Pune, India; 4 Department of Clinical Pharmacology, Nair Hospital, Mumbai, India; 5 Medical Research Council Human Nutrition Research, Elsie Widdowson Laboratory, Cambridge, UK; 6 Apnalaya, Mumbai, India; 7 Public Health Nutrition, University of Southampton, Southampton, UK

**Keywords:** Food-based interventions, India, Micronutrient status

## Abstract

Intakes of micronutrient-rich foods are low among Indian women of reproductive age. We investigated whether consumption of a food-based micronutrient-rich snack increased markers of blood micronutrient concentrations when compared with a control snack. Non-pregnant women (*n* 222) aged 14–35 years living in a Mumbai slum were randomised to receive a treatment snack (containing green leafy vegetables, dried fruit and whole milk powder), or a control snack containing foods of low micronutrient content such as wheat flour, potato and tapioca. The snacks were consumed under observation 6 d per week for 12 weeks, compliance was recorded, and blood was collected at 0 and 12 weeks. Food-frequency data were collected at both time points. Compliance (defined as the proportion of women who consumed ≥ 3 snacks/week) was >85 % in both groups. We assessed the effects of group allocation on 12-week nutrient concentrations using ANCOVA models with respective 0-week concentrations, BMI, compliance, standard of living, fruit and green leafy vegetable consumption and use of synthetic nutrients as covariates. The treatment snack significantly increased β-carotene concentrations (treatment effect: 47·1 nmol/l, 95 % CI 6·5, 87·7). There was no effect of group allocation on concentrations of ferritin, retinol, ascorbate, folate or vitamin B_12_. The present study shows that locally sourced foods can be made into acceptable snacks that may increase serum β-carotene concentrations among women of reproductive age. However, no increase in circulating concentrations of the other nutrients measured was observed.

Multiple micronutrient deficiencies are prevalent among women of reproductive age in India^(^ ^1^ ^–^ ^3^ ^)^. There are limited data available on the micronutrient status of women living in Indian slums. However, there is evidence that such women are at risk of deficiency. A recent review^(^ ^4^ ^)^ of studies investigating the intake of micronutrients among women of reproductive age in low- and middle-income countries has found that in Asia, intakes of several micronutrients were frequently below the WHO/FAO Estimated Average Requirement (EAR)^(^ ^5^ ^)^. The majority of studies reviewed have found that intakes of Fe, folate and vitamin B_12_ were less than half of the EAR, and between 25 and 77 % of women in Asia had mean intakes of vitamin A and vitamin C below the EAR. No data on carotenoid intakes were reported in the review. The authors suggested that these findings may reflect the cereal-based diets that are commonly consumed in such populations, which tend to be low in fruit, vegetables and animal foods. Low micronutrient intakes have adverse implications for women's risk of anaemia, infectious disease and chronic conditions such as cancer. Optimal offspring health is also dependent on adequate maternal nutrition.

Animal foods are relatively expensive, ^(^ ^6^ ^)^ and a large proportion of the Indian population are vegetarian. Milk, fruit and green leafy vegetables are a good source of micronutrients, but intakes of these foods are low, with national survey data indicating that in 2005–6 over 50 % of Indian women consumed fruit less than once per week^(^ ^7^ ^)^. When these data were broken down by region and demographic group, over two-thirds of women living in Mumbai slum areas (*n* 803) consumed green leafy vegetables on a daily basis. However, only frequency data were collected in the survey, and several Indian preparations contain very small amounts of green leafy vegetables ( < 5 g) such as coriander for seasoning. It is, therefore, possible that the proportion of women who consumed a 100 g portion daily as recommended in the Indian National Institute of Nutrition guidelines^(^ ^8^ ^)^ would be considerably smaller than the proportion cited above. A quarter of women reported consuming fruit daily, and approximately 40 % ate fruit less than once per week. Over 15 % of women never consumed milk or curd (yogurt), and 56 % consumed these items less than once per week. Recent quantitative data on dietary intake collected from women living in North Indian slums show that intakes of fruit and vegetables were very low^(^ ^9^ ^,^ ^10^ ^)^. Anand *et al.* ^(^ ^10^ ^)^ found that in slum areas of Haryana state, the mean number of servings per d of fruit and vegetables was 2·2 for women, with only 5·4 % of women consuming five servings per d.

In the present study, we aimed to assess the effects of daily consumption of a food-based intervention in the form of a snack food containing green leafy vegetables, dried fruit and milk powder on blood micronutrient concentrations in non-pregnant, low-income, Indian women of reproductive age.

## Methods

The present study was conducted between October 2009 and March 2010, and was an adjunct to a larger ongoing randomised controlled trial, namely the Mumbai Maternal Nutrition Project (MMNP)^(^ ^11^ ^,^ ^12^ ^)^, which was launched in January 2006 (trial registration: ISRCTN 62811278). The MMNP intervention was based on the results of the Pune Maternal Nutrition Study, an observational investigation in a rural area near the city of Pune, which reported that maternal intakes of green leafy vegetables, fruit and milk during pregnancy were positively associated with offspring birth size measurements^(^ ^13^ ^)^. The MMNP was designed to investigate the effect of maternal consumption of a snack containing green leafy vegetables, fruit and milk for at least 3 months before conception and throughout pregnancy on infant size and mortality. The treatment snack was designed in such a way that in addition to the women's habitual diet, a daily intake of ≥ 75th centile of green leafy vegetables (25 g), fruit (10 g) and milk (12 g) as reported in the Pune Maternal Nutrition Study would be achieved.

The present study was a randomised controlled trial of the MMNP intervention model, and was undertaken to examine its effects on micronutrient status among a separate group of non-pregnant women. Baseline status was not assessed in the main MMNP trial in order to minimise blood collection which may have been a deterrent to participation. The present study was conducted approximately 10 miles away from the MMNP study area, and the participants were not enrolled in the MMNP at any time.

The present study was conducted according to the guidelines laid down in the Declaration of Helsinki, and all procedures involving human subjects were approved by the JJ Hospital Ethics Committee, Mumbai.

### Participants and settings

The participants lived in the Shivaji Nagar slum area of Mumbai with inadequate access to safe water and sanitation, poor structural quality of housing and overcrowding. These criteria have been used by the UN to define slums^(^ ^14^ ^)^. Women were not eligible for the study if they were < 14 or >35 years of age, or reported that they were pregnant or breast-feeding at the time of enrolment. As there is a significant migration rate in the study area, we asked women to enrol only if they intended to remain in the study area for at least 3 months. A power calculation showed that a sample size of eighty-two per group was necessary to demonstrate a difference in the proportion of vitamin A deficiency (defined as serum retinol < 0·7 μmol/l) of the order of 35 % in the control group *v*. 10 % in the treatment group at 90 % power and at the 0·05 significance level^(^ ^15^ ^)^. These estimates were based on data from the WHO Vitamin and Mineral Information System database, whereby the prevalence of vitamin A deficiency ranged from 33 to 35 % in a study of 150 women in urban Calcutta^(^ ^16^ ^)^. Assuming a dropout rate of 20 %, our target sample was 102 in each group.

### Procedure

In October 2009, women living in the Shivaji Nagar area were invited to community meetings at which they were informed about the study by the research team. Written informed consent was obtained from all women who agreed to participate. Those who met the inclusion criteria (*n* 222) were enrolled and randomised to the treatment or control arm of the study. Randomisation was stratified by age and BMI to ensure that the groups did not differ by these variables. The treatment and control snacks were outwardly similar, but their contents were different. To achieve a degree of allocation concealment, we created two treatment and two control groups, each with an independent set of recipes. The women were given colour-coded identity cards based on group allocation. There were no differences in the amounts and types of green leafy vegetables, fruit and milk powder in the treatment snacks. A total of four different snacks were produced daily, in an unpredictable pattern. The snacks were packaged in colour-coded bags to match the women's identity cards and ensure that they were correctly distributed. The staff who measured outcomes were blinded to the women's allocation groups. The two treatment groups and the two control groups were merged for analysis.

Baseline data and venous blood samples were collected before the start of supplementation and within 7 d of enrolment. Demographic data and information on synthetic micronutrient supplement intake in the form of tablets or tonics were collected by means of a questionnaire. A 221-item interviewer-administered FFQ developed for this sample^(^ ^11^ ^)^ was used to collect data on the past week's green leafy vegetable and fruit intake at baseline and at 12 weeks. This was in order to assess any changes in intakes of these foods over the supplementation period. Height was measured to the nearest millimetre using a portable stadiometer (Seca), and weight to the nearest 100 g using electronic scales (Salter). BMI was expressed as kg/m^2^.

During the 12-week supplementation period, women were asked to visit a local community centre 6 d per week (Monday to Saturday) to receive the snacks and consume them under observation. The centre was ≤ 10 minutes' walk from the women's homes. Consumption of the snacks was recorded by a project health worker. Compliance with the intervention was defined as consumption of a mean of ≥ 3 supplements per week over the 12-week period. Follow-up blood samples were collected at 12 weeks after the beginning of supplementation. Women who were found to be moderately or severely anaemic (Hb < 100 g/l) at baseline or at 12 weeks were offered Fe tablets. Women who were mildly anaemic (Hb 100–119·9 g/l) were informed of their status, and advised to consult a doctor and consider taking Fe supplements. Anaemic women were eligible to remain in the study.

### Intervention

Women were given one cooked snack per d such as a ‘samosa’ or ‘patty’ made from locally available food ingredients. The treatment snacks were designed to provide women with 25 g fresh green leafy vegetables (e.g. spinach, *Colocasia*, coriander and fenugreek leaves), 10 g dried fruit (e.g. Figs, dates and raisins) and 12 g whole milk powder in addition to their habitual diet. The control snacks contained foods, such as potato, sago or tapioca. Both types of snacks were prepared with binding ingredients, such as wheat or maize flour, to which spices were added. The snacks were cooked by shallow frying in sunflower oil. Several varieties of the treatment (*n* 5) and control snacks (*n* 7) were developed, to prevent monotony. The average weight of the snacks was approximately 65 g (treatment) and 36 g (control). The women were advised to consume the snacks in addition to their habitual diet. One snack per d was given to the women at a time least likely to interfere with their usual intake, between 15.00 and 18.00 hours, Monday to Saturday.

### Nutrient content of the intervention

Before nutrient analysis, the snacks were frozen in Mumbai and transported on dry ice to the UK, where they were analysed at Eclipse Laboratories (Cambridge, UK) for micronutrient content. Over 75 % of the recipes were analysed and mean micronutrient content was derived (Table 1).

**Table 1:** Mean nutrient composition and mean percentage contribution to nutrient requirements of the treatment and control snacks* (Mean values and standard deviations)

	Nutrient content
	Energy† (MJ)	Fe (mg)	RE (μg)	Vitamin C (mg)	Folate‡ (μg)	Vitamin B_12_ (μg)
	Mean	sd	Mean	sd	Mean	sd	Mean	sd	Mean	sd	Mean	sd
Treatment snacks (mean weight 65 g)												
Nutrient content per serving	0·61	0·07	3·9	1·3	141	85	2·1	3·0	67·5	30·6	0·31	0·13
Percentage of RNI§ per serving			20	18	4	11	12
Control snacks (mean weight 36 g)												
Nutrient content per serving	0·37	0·05	0·9	0·26	2	1	0·0	0·0	6·1	4·6	0·18	0·25
Percentage of RNI§ per serving			5	< 1	< 1	1	7

RE, retinol equivalents; RNI, reference nutrient intake.

* Weighted average based on the number of days that the snacks were distributed over the study period.

† Energy content was calculated from the Indian Food Composition Tables^(^ ^17^ ^)^.

‡ Total folate.

§ FAO/WHO-recommended reference nutrient intake during the first trimester of pregnancy^(^ ^5^ ^)^.

To assess mineral content, samples were dried and ashed at 550°C for 16 h, then dissolved in 5 m-HCl, and scandium (internal standard)/caesium chloride solution was added. After filtration and dilution to known volumes with water, the concentration of each mineral was determined by ‘Liberty series II’ inductively coupled plasma atomic emission spectrometry.

Ascorbic acid was extracted from the sample using metaphosphoric acid and EDTA. Ascorbic acid was then enzymatically oxidised to dehydroascorbic acid, which was condensed with *O*-phenylenediamine to give the fluorescent quinoxaline derivative. The latter was separated from interfering compounds by reverse-phase HPLC with fluorometric detection.

β-Carotene analysis was conducted away from natural light using amber glassware. The sample was saponified for 30 min at 95°C with ethanolic potassium hydroxide, and carotene was extracted with hexane. Hexane was evaporated to dryness and carotene dissolved in the mobile phase and quantified by HPLC with UV detection, against a calibration standard of known concentration.

Folates were extracted from the samples using 0·1 m-potassium phosphate buffer and by heating for 15 min at 100°C. The filtrate was diluted to a suitable level and treated with deconjugase enzyme. l-Ascorbic acid was also added to prevent oxidation. This complex was incubated at 37°C for 4 h. Sample extracts were diluted to values within the calibrated range. Folic acid casei media were added to the diluted samples, which were then covered with aluminium foil and sterilised at 121°C in an autoclave. The assay was inoculated with *Lactobacillus rhamnosus* and incubated overnight at 37°C. The concentration of folate in the sample was measured spectrophotometrically.

The energy content of the snacks was calculated using the values for raw ingredients from Indian Food Composition Tables^(^ ^17^ ^)^.

### Biochemical measurements

Within 1 h from collection, venous blood was centrifuged at 20°C for 10 min (REMY). Depending on the assay, plasma or serum was pipetted into vials and kept on dry ice for up to 8 h before being transported to a − 80°C freezer for storage until analysis. For vitamin C analysis, 0·3 ml plasma was added to 0·3 ml of 10 % metaphosphoric acid and stored at − 80°C until analysis.

A quantitative test kit based on solid-phase ELISA was used to measure serum ferritin concentrations (ELISA; Diagnostic Automation, Inc.). The system used one anti-ferritin antibody for solid-phase (microtitre wells) immobilisation and another mouse monoclonal anti-ferritin antibody in the antibody–enzyme (horseradish peroxidase) conjugate solution. The test sample was allowed to react simultaneously with the antibodies, resulting in the ferritin molecules being sandwiched between the solid phase and enzyme-linked antibodies. After incubation at room temperature for 60 min, the wells were washed with water to remove unbound antibodies. A solution of tetramethylbenzidine was added and incubated for 20 min, 2 m-HCl was added, and ferritin concentration was measured spectrophotometrically at 450 nm.

For the measurement of β-carotene, thawed subsamples of plasma were extracted with *n*-hexane (containing butylated hydroxytoluene) in the presence of aqueous SDS and absolute ethanol (the latter containing α-tocopherol acetate (internal standard)). The upper organic phase was evaporated to dryness under vacuum and then redissolved in 250 μl of the mobile phase. Aliquots (50 μl) were then injected onto a 3 μm YMC-Pack Pro C_18_ column. The mobile phase was acetonitrile (44 %), methanol (44 %) and dichloromethane (12 %), by volume. For each sample, β-carotene was measured at 450 nm using an Empower 2-controlled HPLC system (Waters Ltd), with a photodiode array detector. Simultaneously, the internal standard was measured at 284 nm, and the ratio of the responses calculated (response factor). β-Carotene calibration lines were obtained from semi-pure, commercially available β-carotene. The concentration of the calibrants was determined from their absolute optical densities and known extinction coefficient. These were then corrected to 100 % purity, by means of their HPLC purity profiles^(^ ^18^ ^)^.

Retinol levels were determined using a standard HPLC kit (Recipe Chemicals+Instruments, GmbH). Sample preparation involved two steps: (1) extraction and (2) stabilisation. First, 150 μl of calibrator/control/serum and 150 μl of precipitant P (containing internal standard) were mixed in a sample preparation vial, and then centrifuged at 10 000 ***g*** for 5 min. Second, stabilisation was performed by mixing 100 μl of the centrifuged supernatant and 100 μl cooled stabilising reagent S followed by centrifugation at 10 000 ***g*** for 5 min. A 50 μl supernatant was injected into the Thermo Spectra Isocratic HPLC System with a UV detector (Thermo Fisher Scientific Private Limited). Injection volume was 50 μl with a flow rate of 1·5 ml/min. The initial wavelength was 325 nm and after 3·5 min, the wavelength was altered to 295 nm. The column temperature was maintained at 30°C. The retention time for retinol was within 2 min and for the internal standard was 5 min. The limit of detection was 10 ng/ml, and the limit of quantification 20 ng/ml. For validation of data, ClinChek^®^ Serum Control Levels I and II (Recipe Chemicals+Instruments, GmbH) were used along with each batch of samples^(^ ^19^ ^)^.

Ascorbic acid concentrations were assayed using an in-house method based on the procedure described by Vuilleumier & Keck^(^ ^20^ ^)^, adapted for microplates. Ascorbic acid in the metaphosphoric acid-stabilised plasma sample was converted into dehydroascorbic acid by ascorbate oxidase (Sigma). The resulting dehydroascorbate was coupled with *O*-phenylenediamine to give a fluorescent quinoxaline. The formation of this quinoxaline was linearly related to the amount of vitamin C in the sample, over the range of 0–10 μg/ml (0–5 μm). Fluorescence was measured using a BMG LABTECH FLUOstar OPTIMA plate reader. The accuracy of the fluorometric assay procedure used was ascertained by participation in the external quality assessment programme, provided by the National Institute of Science and Technology, USA, which validates results against Standard Reference Preparation no. 970.

Plasma folate concentrations were measured by microbiological assay using a chloramphenicol-resistant strain of *Lactobacillus* *casei*, and using Victor-2 (PerkinElmer Life Science)^(^ ^21^ ^,^ ^22^ ^)^. Plasma vitamin B_12_ was measured by microbiological assay using a colistin sulphate-resistant strain of *Lactobacillus* *leichmannii* ^(^ ^23^ ^,^ ^24^ ^)^.

Plasma C-reactive protein (CRP) was measured to assess levels of acute-phase reactants to interpret the results of serum ferritin and retinol analysis. CRP was assayed using a high-sensitivity ELISA kit (United Biotech), and using Victor-2 (PerkinElmer Life Science). The CV for the assay was < 11 %. The cut-off value for active inflammation was 5000 ng/ml^(^ ^25^ ^)^.

### Statistical analysis

Where data were normally distributed, means and standard deviations are presented, and elsewhere medians and interquartile ranges (IQR) are given. BMI was log-transformed for regression analysis. Independent *t* tests and χ^2^ tests were used to assess the differences in baseline characteristics between intervention groups. Paired *t* tests were used to assess within-group changes in fruit and green leafy vegetable consumption over the study period. Independent *t* tests were used to assess the differences between groups with respect to the change in fruit and green leafy vegetable consumption at baseline and at 12 weeks. ANCOVA models for each nutrient were used to assess treatment effects with 12-week concentrations as dependent variables and respective baseline concentration, age, BMI, standard of living index, compliance with supplementation, use of nutritional supplements, 12-week fruit intake frequency and 12-week green leafy vegetable intake frequency as covariates.

In the case of ferritin and retinol, women with CRP concentrations >5000 ng/ml were excluded from the analyses, ^(^ ^26^ ^,^ ^27^ ^)^ and the models were adjusted for plasma CRP concentrations. All statistical analyses were performed using the SPSS software package version 19 (SPSS, Inc.).

## Results

### Participant characteristics

Table 2 presents the anthropometric and demographic characteristics of the women enrolled in the trial, as well as the proportion of women who were nutrient deficient at baseline. A large proportion of the women in the study were underweight and chronically energy deficient based on BMI. The majority of women were of Muslim faith. Over three-quarters of the women had completed at least secondary level education. A third of them were in paid employment at the time of the study. Our data indicate that the majority of the women were Fe deficient, about one-fifth were retinol deficient (using the 1·047 μmol/l cut off) and two-fifths were vitamin C deficient. Fewer than 10 % had low folate or vitamin B_12_ status. Almost two-thirds were mildly anaemic, and very few severely anaemic. There were no significant differences in any of the baseline characteristics between the control and treatment groups.

**Table 2:** Baseline characteristics of the women by intervention group (all 222 women enrolled) (Mean values and standard deviations; medians and interquartile ranges (IQR); number of participants and percentages)

	Control (*n* 114)	Treatment (*n* 108)	
	Median	IQR	Median	IQR	*P* *
Age (years)	20	17–25	21	17–27	0·999
Weight (kg)	41·6	35·7–46·8	40·0	36·7–46·8	0·477
Height (cm)					0·750
Mean	149·6	149·5	
sd	5·7	5·6	
BMI (kg/m^2^)	18·6	16·3–20·1	18·4	16·4–20·5	0·626
	*n*	%	*n*	%	
Religion					0·593
Hindu	20	17·5	14	13·0	
Muslim	90	78·9	91	84·3	
Other	4	3·5	3	2·8	
Education					0·181
Primary or less	27	23·7	19	17·6	
Secondary	80	70·2	78	72·2	
Graduate	7	6·2	5	4·6	
Occupation					0·587
Semi-skilled/unskilled	27	23·7	25	23·1	
Skilled/self-employed	13	11·4	6	5·6	
Professional	4	3·5	2	1·9	
Not working	70	61·4	75	69·4	
Deficiency status					
Ferritin ( < 15 ng/ml)†	100	87·7	85	78·7	0·258
Ferritin ( < 15 ng/ml)‡	61	88·4	55	84·6	0·520
Retinol ( < 1·05 μmol/l)	20	18·5	24	22·2	0·360
Retinol ( < 1·05 μmol/l)‡	17	19·1	16	19·0	0·972
Ascorbate ( < 11 μmol/l)	46	40·4	38	35·1	0·385
Folate ( < 6·8 nmol/l)	9	7·9	9	8·3	0·805
Vitamin B_12_ ( < 150 pmol/l)	3	2·6	2	1·9	0·250
Hb ( < 120 g/l)	69	60·5	71	65·7	0·844
Hb ( < 80 g/l)	3	2·6	1	0·9	0·346

* *P* value relates to differences between the groups based on *t* tests for continuous variables (values were log-transformed when distributions were not normal) and χ^2^ tests for categorical variables.

† Includes all women.

‡ Excludes women with C-reactive protein concentrations >5000 ng/ml.

During the study, thirty women reported taking synthetic micronutrient supplements. Of these, eighteen took multiple micronutrient supplements, eight took Fe and folate, and four took Fe. The median monthly family income was 4000 (IQR 3000–5000) rupees equivalent to approximately USD88 (IQR USD66–110), and the median family size was six (IQR 5–7) persons. Approximately 15 % of the women lived in ‘katcha’-style housing made from plastic sheeting, sticks and textiles. The remainder had ‘semi-pucca’ or ‘pucca’ walls and floors, made with cement and corrugated metal roofs. Most women (86 %) used a shared pit toilet.

### Participant flow

**Fig. 1:**
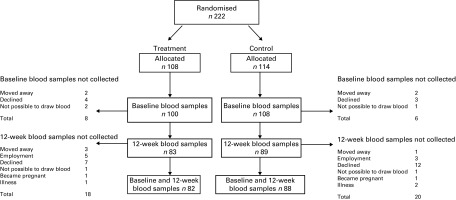
Flow chart of the study participants.

Of the 222 women randomised, a total of 208 women with baseline blood measurements started receiving the intervention (Fig. 1). Of these, blood was collected at 12 weeks from 172 women and blood samples were available at both time points for 170 women. Compliance (defined as the proportion of women who consumed ≥ 3 snacks per week) was 85 % in the treatment and 90 % in the control groups. The median weekly snack consumption of those in the final analysis was 5·0 (IQR 4·4–5·5) in the control group and 4·9 (IQR 3·8–5·3) in the treatment group.

### Dietary intake

Habitual fruit intake increased in both groups: from a mean of 2·1 (sd 3·1) to 3·9 (sd 5·5) servings per week in the control group (*P*= 0·001), and from 2·0 (sd 3·1) to 3·8 (sd 5·1) servings per week in the treatment group (*P*= 0·003). There was no difference between the groups in terms of the change in fruit intake (*P*= 0·987). Habitual intakes of green leafy vegetables in the control group increased by a mean of 0·8 (sd 2·2) servings per week from 1·3 (sd 1·4) to 2·1 (sd 2·0) servings per week (*P*< 0·001). There was no difference between 0-week and 12-week green leafy vegetable intakes in the treatment group. Mean intakes were 1·4 (sd 1·7) servings per week at baseline and 1·7 (sd 2·6) at 12 weeks (*P*= 0·420). The difference between the groups in terms of the change in intakes over the study period did not reach any statistical significance (*P*= 0·144; data not shown).

### Blood micronutrient concentrations

There was no effect of the treatment on serum ferritin concentrations (Table 3). When we excluded the women whose CRP levels were >5000 ng/ml (*n* 28) and reran the analysis, there was little difference in the median ferritin concentrations observed. The data indicated a statistically significant treatment effect on β-carotene concentrations of 47 nmol/l. This was approximately 12 % of the baseline concentrations. The CV of the β-carotene assay was approximately 15 %. No effect of group allocation was observed for retinol, ascorbate, folate or vitamin B_12_.

**Table 3:** Blood nutrient concentrations at baseline and at 12 weeks of supplementation and the effects of treatment on blood nutrient concentrations at 12 weeks (Medians and interquartile ranges (IQR); *B* values and 95 % confidence intervals)

	Control	Treatment			
		0 weeks	12 weeks		0 weeks	12 weeks			
	*n*	Median	IQR	Median	IQR	*n*	Median	IQR	Median	IQR	*B* (treatment effect)*	95 % CI	*P* †
Ferritin (μmol/l)	83	6·4	4·6–11·0	6·4	5·0–11·5	77	6·4	5·0–11·8	7·6	5·0–10·7	− 0·12	− 0·01, 0·26	0·074
Ferritin (μmol/l)‡	69	6·2	4·6–11·0	6·4	4·8–11·5	66	6·4	5·0–12·0	7·6	5·0–11·0	− 0·12	− 0·03, 0·27	0·128
β-Carotene (nmol/l)	85	390	305–470	440	340–540	80	385	323–470	470	380–610	47·1	6·5, 87·7	0·023
Retinol (ng/ml)	83	409	326–490	395	324–476	79	378	297–484	358	291–474	− 5·2	− 30·7, 41·0	0·777
Retinol (ng/ml)‡	69	421	319–537	396	324–466	68	375	309–485	367	291–476	1·0	− 38·7, 36·6	0·956
Ascorbate (μmol/l)	66	12·5	8·8–20·3	14·6	9·6–27·0	70	12·7	8·6–22·3	17·0	9·9–29·5	0·17	− 4·76, 5·10	0·947
Folate (nmol/l)	80	14·1	9·0–21·4	13·1	9·2–18·4	77	13·3	9·9–19·6	13·3	9·2–17·5	− 2·81	− 10·90, 5·28	0·755
Vitamin B_12_ (μmol/l)	79	250	178–351	255	187–304	76	290	228–340	259	208–326	19·02	− 11·61, 49·67	0·222
Hb (g/l)	88	114	103–125	119	106–126	82	114	105–122	117	108–123	− 1.60	− 4·90, 1·70	0·340

* Treatment effects were estimated using ANCOVA models with the following covariates: baseline concentrations; age; BMI; compliance with supplementation protocol; use of micronutrient tablets; fruit intake at visit 3; green leafy vegetable intake at visit 3. Where ferritin and retinol concentrations were outcomes, C-reactive protein concentrations were included in the models.

† *P* value relates to ANCOVA tests assessing the effects of the treatment groups on 12-week blood nutrient concentrations adjusted for baseline concentrations, age, BMI, compliance with supplementation protocol, use of micronutrient tablets, fruit intake at visit 3, and green leafy vegetable intake at visit 3.

‡ Excluding women with plasma C-reactive protein concentrations >5000 ng/ml.

## Discussion

The present study aimed to assess the effects of consumption of a micronutrient-rich food-based intervention on the nutritional status of low-income group Indian women. We found that consumption of the treatment snacks for 12 weeks was associated with a small increase in β-carotene concentrations relative to the control group. The mean difference in the change between groups was 47 nmol/l, which was just over 12 % of baseline β-carotene values. The CV of the assay was of a similar magnitude, but due to the randomised design, it could be reasonably expected that the measurement error would not differ between the groups. There was no significant effect of the intervention allocation on the change in the concentrations of any of the other micronutrients studied.

In the UK, the effect of adding 85 g/d of raw watercress to the usual diet for 8 weeks was an increase in β-carotene levels by a mean of 100 nmol/l^(^ ^28^ ^)^. Considering the quantity of green leafy vegetables in the intervention in the present study, it would appear that the effect size is consistent with this finding.

The treatment effect in the present study was considerably smaller than that in studies where alterations to the entire dietary pattern such as increasing daily intake of fruit and vegetables from two to ten portions per d were implemented. Such interventions have achieved up to a fivefold increase in β-carotene concentrations^(^ ^29^ ^–^ ^31^ ^)^. It is questionable how sustainable such changes to diets would be in the Indian slum population.

There are inconsistent effects of vitamin A-rich foods on nutritional status among populations in Asia and Africa. Among Bangladeshi men, vitamin A pool size was increased following a spinach intervention^(^ ^32^ ^)^. Among Kenyan children, it has been reported that dehydrated amaranth and cowpea leaves increased serum β-carotene and retinol concentrations^(^ ^33^ ^)^. However, a recent study in Bangladesh assessed the effect of the consumption of bio-fortified orange-fleshed sweet potato on vitamin A pool size among vitamin A-deficient women living in a low-income area of Dhaka city^(^ ^34^ ^)^. The potato intervention increased circulating β-carotene concentrations, but there was no effect on vitamin A stores. This finding indicated that the conversion of β-carotene to vitamin A was limited in this population. The authors concluded that protein deficiencies among these women may have led to reduced synthesis of BCMO1 (β-carotene 15,15′-monooxygenase 1), which, in turn, would reduce the conversion of β-carotene to retinol.

Over three-quarters of the women in the present study had retinol and folate concentrations above the cut-offs for deficiency. Therefore, it is possible that the majority of the women in this sample were not deficient in these nutrients. In addition, the duration of the supplementation period may have been too short for retinol status to be altered. It was necessary for us to achieve a balance between resources, compliance with the protocol and observing an effect of the supplement on micronutrient status. Furthermore, in the case of vitamin C, the content in the snacks was low, probably due to the cooking process. There was some experimentation with different methods of cooking, and preparing the snacks to preserve vitamin C content, but the most acceptable method to the women was shallow frying, and this method was used, as it was important to achieve adherence to the protocol.

A study on a small group of healthy young females in Pune, West India, compared the short-term effects of consumption of a green leafy vegetable meal with a standard meal without green leafy vegetables^(^ ^35^ ^)^. No difference in plasma β-carotene or vitamin C concentrations between the two groups was observed 4 h after the meal. However, after a 3-week intervention period constituting daily supplementation with 100 g cooked green leafy vegetables and 10 g oil, there was a significant increase in plasma concentrations of both nutrients. The authors concluded that an intake of 100 g green leafy vegetables per d plus 10 g oil could be an effective strategy for improving micronutrient status of young Indian women. The treatment snacks in the present study contained approximately 25 g green leafy vegetables per serving; it is possible that there is a ‘threshold nutrient intake’ above which a change in nutrient concentration would be observed. For example, consumption of four snacks per d may have led to significant increases in the concentrations of the other nutrients, and a more marked increase in β-carotene concentrations in the treatment group.

We assessed the possible impact of inflammation on ferritin status using CRP concentrations; however, this did not alter the lack of association with treatment allocation. The snacks contained almost 4 mg Fe per serving. However, it is likely that there was insufficient bioavailable Fe in the snacks due to the inhibition of Fe uptake, attributable, in turn, to frequent consumption of phytate-containing cereal-based foods, tea containing polyphenols, milk protein and polyphenols in green leafy vegetables in the snacks themselves.

The present study had several strengths and limitations. Before the study, there was a paucity of data on the effect of long-term food-based interventions on the nutritional status of women in low-income settings. We used a randomised controlled design, but as with many food-based intervention studies, it was not possible to blind the intervention. It is unlikely that this would have influenced the findings, given that the outcomes were objectively assessed, and the group allocations were not known to the laboratory staff who made the nutrient concentration measurements. A strength of the study is that all of the participants started and completed the intervention at the same time, thus creating an internal control for seasonal changes in food intakes.

The sample size calculation was based on serum retinol concentrations, as this was the nutrient for which data were available for the Indian population. Based on previously published data, we had perhaps overestimated the proportion of women who would be retinol deficient, and it is possible that the study was underpowered for other nutrients, for which no status data of this sample were available. It would have been of interest to have measured the change in Zn status, as green leafy vegetables are a source of this mineral, but circulating Zn concentrations are tightly regulated, and this would have required a different methodology which was not feasible within the present study.

It is possible that the women might have changed their habitual diet on account of being enrolled in the study; it is also conceivable that women in the treatment group reduced their consumption of green leafy vegetables because they were aware of the snack intervention. Women in the control group may have done the opposite, thus negating the effect of the snacks on micronutrient status. Our data suggest that this did happen to a certain extent in the present study, and the effect size that we have shown in relation to β-carotene concentrations may be an underestimation. A similar phenomenon was observed in the UK school fruit scheme, whereby children who received fruit at school were given less fruit at home, and so an intervention designed to increase children's intake of fruit by one portion per d resulted in only a 0·5 portion per d increase^(^ ^36^ ^)^, and this was perhaps due to the parents' assumption that children required less fruit at home in the context of the school fruit scheme^(^ ^37^ ^)^. This phenomenon poses a crucial challenge to the design of food-based interventions.

In conclusion, the present study indicated that small changes in intakes of fruit, green leafy vegetables and milk in a food-based snack intervention might have led to increased levels of circulating β-carotene concentrations in the sample chosen.
